# Radium Therapeutics

**Published:** 1914-03

**Authors:** 


**Radium Therapeutics.** By N. S. Finzi, M.B. Lond. Pp. ii, 112. London: Henry Frowde and Hodder and Stoughton. 1913. 6s. net.

A monograph by one of the most experienced pioneers in such a new branch of therapy as this work expounds is difficult to criticise, but one notable and attractive feature is the author's diffidence in making claims for radium therapy, particularly in diseases which in his own experience it has proved of doubtful value. The introductory chapters on the nature of the various radiations, the different methods by which they can be utilised, and on the histological actions of the different rays, will be very much appreciated by those who have had but little practical acquaintance with the subject. The succeeding sections dealing with the diseases in which radium has been employed clinically will be of the utmost value to those who have patients under their care for whom it is thought radium therapy presents useful possibilities.

